# Propranolol as Salvage Therapy for Refractory Congenital Chylous Ascites in a Preterm Neonate: A Case Report and Review of the Literature

**DOI:** 10.3390/children13091141

**Published:** 2026-08-26

**Authors:** Nicoletta Menzella, Francesca Riitano, Simonetta Costa, Francesca Paola Fusco, Simona Fattore, Chiara Tirone, Francesca Stollagli, Giovanni Vento

**Affiliations:** 1 Division of Neonatology, Department of Women and Child Health, Fondazione Policlinico Universitario A. Gemelli IRCCS, 00168 Rome, Italy; francescapaola.fusco@policlinicogemelli.it (F.P.F.); simona.fattore@guest.policlinicogemelli.it (S.F.); chiara.tirone@policlinicogemelli.it (C.T.); giovanni.vento@unicatt.it (G.V.); 2 Neonatal Intensive Care Unit, Fondazione Policlinico Universitario “Agostino Gemelli” IRCCS, Largo Agostino Gemelli, 8, 00168 Rome, Italy; 3 Neonatology and Neonatal Intensive Care Unit, Policlinico Casilino, 00169 Rome, Italy; scosta@policlinicocasilino.it; 4 Neonatal Intensive Care Unit, Ospedale Policlinico Casilino, Via Casilina, 1049, 00169 Rome, Italy; 5 Obstetrics and Obstetric Pathology Unit, Department of Women and Child Health, Fondazione Policlinico Universitario A. Gemelli IRCCS, 00168 Rome, Italy; francesca.stollagli@policlinicogemelli.it; 6 Department of Women and Child Health Sciences, Child Health Area, Università Cattolica Del Sacro Cuore, Largo F. Vito 1, 00168 Rome, Italy

**Keywords:** congenital chylous ascites, propranolol, beta-adrenergic blocker, neonate, conservative therapy, case report

## Abstract

**Highlights:**

**What are the main findings?**

• Oral propranolol was associated with resolution of refractory congenital chylous ascites in a preterm neonate who had not responded to fasting, total parenteral nutrition, and maximal-dose octreotide.
• A review of the literature identified nine additional neonates with heterogeneous lymphatic flow disorders, not limited to congenital chylous ascites, successfully treated with propranolol, suggesting a possible therapeutic benefit across cases.

**What are the implications of the main findings?**

• Propranolol may represent a potential salvage therapy option for neonates with congenital chylous ascites refractory to conventional conservative management and octreotide, though its effect could not be isolated from concurrent octreotide treatment in this case.
• Prospective multicenter studies are needed to define optimal dosing and treatment duration for propranolol in neonatal lymphatic flow disorders.

**Abstract:**

**Background/Objectives:** Congenital chylous ascites is a rare neonatal condition characterized by the accumulation of lymphatic fluid within the peritoneal cavity. The disorder can be difficult to manage, as many infants show limited response to standard conservative strategies such as dietary modification and supportive care. Currently, no universally accepted therapeutic guidelines exist. We report the successful use of propranolol in the management of refractory congenital chylous ascites. **Methods:** Clinical data were collected from a 35-week preterm male infant who had prenatal ultrasound evidence of fetal ascites from the 28th week of gestation. Postnatal management initially included fasting, total parenteral nutrition, and from day 9 of life, continuous infusion of octreotide. Due to the persistent ascites, oral propranolol was added to the therapy on the 31st day of life. Current literature was reviewed to compare our findings with those available. **Results:** Following the addition of propranolol to ongoing octreotide therapy, the infant demonstrated gradual clinical improvement with decreased abdominal distension and reduction of ascitic fluid; as propranolol and octreotide were administered concurrently for approximately 30 days, a causal effect could not be isolated to propranolol alone. Oral propranolol was administered at 0.5 mg/kg every 6 h (2 mg/kg/day total), with heart rate, blood pressure, and glucose monitored throughout treatment; no propranolol-related adverse events (bradycardia, hypotension, bronchospasm, hypoglycemia, or feeding intolerance) occurred. Ultrasonographic resolution of ascites was achieved by day 48, the peritoneal drainage catheter having already been removed on day 34; propranolol was subsequently tapered and discontinued by day 68, and the patient was discharged in stable condition on day 93, with no recurrence on serial ultrasound over the 45 days from resolution to discharge. A narrative review of the literature identified nine infants with lymphatic disorders successfully treated with propranolol. **Conclusions:** This case suggests a possible temporal association between propranolol administration and clinical improvement in a neonate with congenital chylous ascites refractory to conventional management; no propranolol-related adverse events were observed during treatment. Given the rarity of this condition and the absence of standardized treatment guidelines, further studies—potentially through multicenter registries or prospective case series—are warranted to establish optimal dosing strategies, evaluate long-term outcomes, and clarify the role of propranolol in this setting.

## 1. Introduction

Lymphatic system disorders in neonates are rare and potentially life-threatening conditions associated with significant morbidity and mortality. These disorders, frequently idiopathic, result from the accumulation of lymphatic fluid in the pleural or peritoneal space due to injury or malformation of the thoracic duct or its branches, leading to retrograde flow and third-space fluid collection. Severe lymphatic abnormalities can cause respiratory compromise, immune suppression, sepsis, hypoalbuminemia, malnutrition, coagulopathy, decreased venous return, and in advanced cases, cardiac failure and hydrops [1,2].

Lymphatic disorders are typically categorized by anatomical site and extent: chylothorax refers to lymph accumulation in the pleural space, and chylous ascites to lymph accumulation in the peritoneal cavity, while central lymphatic flow disorder and generalized lymphatic anomaly describe more diffuse, multi-compartmental involvement of the lymphatic system. Congenital chylous ascites, though rare, is among the most common causes of non-immune ascitic fluid accumulation in neonates [3]. Its pathogenesis is not fully understood, but recent evidence has implicated vascular endothelial growth factor and angiopoietin family members in the development and regulation of the lymphatic system. Antenatal interventions may improve outcomes, although they carry significant procedural risk: thoracentesis and thoracoamniotic shunting are primarily used for pleural effusions, whereas paracentesis is the corresponding intervention for fetal ascites [3,4,5].

Congenital forms are associated with central lymphatic obstruction or developmental anomalies such as lymphatic dysplasia and may occur alone or in conjunction with genetic syndromes including Noonan syndrome, trisomy 21, and Turner syndrome. Acquired forms are usually iatrogenic, following surgery involving the thoracic duct, or secondary to conditions such as central venous thrombosis or tumors [6,7].

To date, no gold standard treatment has been established [1,3]. Conservative postnatal treatment includes fasting followed by dietary modification using defatted human milk or medium-chain triglyceride (MCT) formulas, and octreotide [8]. Octreotide, although commonly used, shows variable efficacy and carries a significant risk of adverse effects. Sirolimus has shown variable success depending on the underlying lymphatic anomaly, while surgical intervention remains a last resort [9]. Recent literature suggests that propranolol may be a viable pharmacological alternative for congenital lymphatic anomalies [8,9]. Its proposed anti-lymphangiogenic and hemodynamic effects, discussed in detail below, provide a plausible rationale for its use specifically in chylous ascites, where excess lymphatic flow and pressure sustain fluid accumulation. While propranolol has previously been reported in a small number of neonates with congenital chylous ascites specifically, its use in this condition remains far less documented than in chylothorax. This report describes the case of a preterm neonate with congenital chylous ascites, refractory to conservative management and octreotide, successfully treated with propranolol, and adds to this limited existing experience.

## 2. Case Report

A 3160 g male infant was born at 35^+3^ weeks^+days^ of gestation via spontaneous vaginal delivery. Fetal ascites was diagnosed prenatally at 28 weeks of gestation and was managed with amnioreduction for associated polyhydramnios and with fetal paracentesis 48 h before delivery. Genetic testing (array comparative genomic hybridization) on the prenatally obtained ascitic fluid excluded rasopathies and chromosomal abnormalities; this prenatal sample was not otherwise biochemically characterized.

Biochemical analysis of fluid obtained postnatally on DOL 0 showed an exudative profile (total protein 31.0 g/L, exceeding the conventional 25–30 g/L threshold distinguishing exudates from transudates), with glucose 56 mg/dL, pH 8.5, creatinine 0.85 mg/dL, albumin 19 g/L, and lactate dehydrogenase 126 U/L. A white blood cell count on the same sample revealed 2.9 × 10^9^/L leukocytes, with 91.8% mononuclear cells and 7.9% polymorphonuclear cells, consistent with our institutional diagnostic threshold for chylous fluid in a fasting patient (leukocyte count > 1000/µL with >70–80% lymphocytes); a repeat leukocyte count on DOL 30 showed 95% mononuclear cells, confirming persistent lymphocytic predominance. A separate metabolic panel on DOL 2 showed ascitic fluid triglycerides of 41 mg/dL, with a same-day paired serum triglyceride level of 60 mg/dL (ascitic-to-serum ratio 0.68). Both values are unremarkable and the ratio does not meet classical biochemical thresholds for chylous fluid; however, this is expected in the fasting state, since dietary chylomicron and triglyceride content of chyle is minimal during fasting, which is why our institutional threshold of ≥110 mg/dL for ascitic fluid triglycerides applies only to patients receiving enteral feeding. Chylomicrons were not specifically assayed.

At birth, the infant showed good cardiorespiratory adaptation with Apgar scores of eight and nine at one and five minutes, respectively. However, due to persistent cyanosis and reduced chest expansion, continuous positive airway pressure and oxygen therapy up to 50% were administered, and the neonate was admitted to the neonatal intensive care unit. The baby had no dysmorphic features but displayed massive abdominal distension. Vital signs remained stable.

Abdominal ultrasound confirmed massive ascites, while thoracic ultrasound excluded pleural effusion. A peritoneal drainage catheter was inserted, yielding approximately 360 mL of citrine fluid. Biochemical analysis confirmed chylous ascites. Bacterial and viral tests on the fluid were negative.

**Figure 1:**
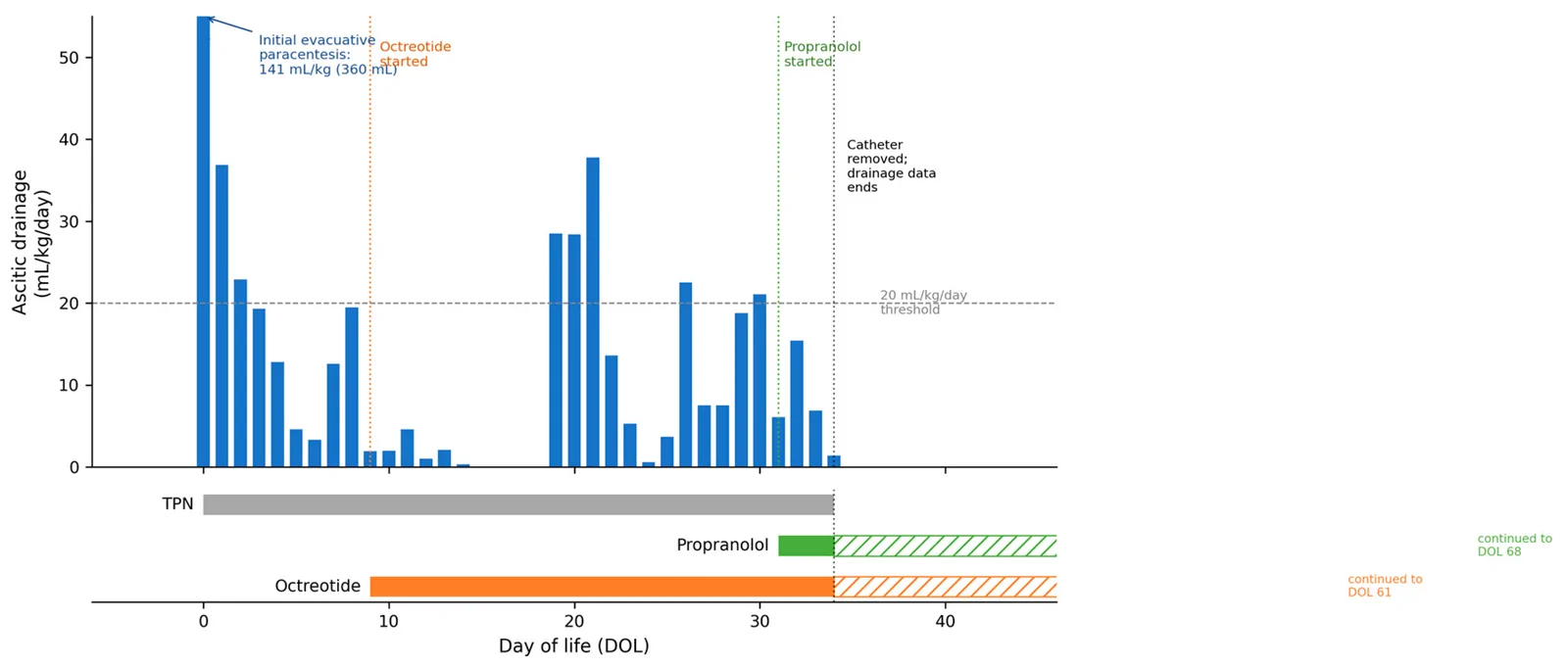
Daily ascitic drainage volume (mL/kg/day) from the peritoneal catheter, DOL 0–34 (until catheter removal), with concurrent therapies shown below (TPN, octreotide, propranolol). The initial value (DOL 0, 141 mL/kg) reflects the single evacuative paracentesis at birth rather than steady-state daily production. The dashed line marks 20 mL/kg/day, the threshold used clinically to define persistent high-output ascites.

The infant was kept nil per os and started on total parenteral nutrition, with intravenous albumin supplementation for hypoalbuminemia secondary to lymphatic protein loss; coagulation studies were monitored serially and remained within acceptable limits, without the need for fresh frozen plasma. Electrolyte disturbances (including transient hyperphosphatemia) were managed through the adjustment of parenteral nutrition composition, and standard fat-soluble vitamin supplementation was provided as part of the parenteral nutrition regimen. Functional echocardiography, performed serially as part of routine monitoring, excluded a structural cardiac cause of the ascites, showing only a small patent ductus arteriosus and patent foramen ovale of no hemodynamic significance. An abdominal Doppler ultrasound, performed later in the course for evaluation of worsening cholestasis, incidentally confirmed a patent portal vein without evidence of thrombosis; dedicated lymphatic imaging (e.g., MR lymphangiography) was not performed. Due to persistent drainage, octreotide infusion was started on day of life (DOL) nine at 1 µg/kg/h and progressively increased to a maximum of 10 µg/kg/h by DOL 26. Because chylous production remained persistently >20 mL/kg/day despite five consecutive days at the maximum octreotide dose (DOL 26–31), this was considered a treatment failure. Contraindications to propranolol (cardiogenic shock, sinus bradycardia greater than first-degree block, reactive airway disease, reduced cardiac contractility, hyperkalemia) were excluded, and oral propranolol at 0.5 mg/kg every 6 h (total 2 mg/kg/day, the maximum dose for preterm neonates per our institutional protocol) was initiated on DOL 31 directly at this dose, without gradual escalation. Heart rate, blood pressure, and glucose levels were monitored throughout treatment for bradycardia, hypotension, bronchospasm, and hypoglycemia, and feeding tolerance was monitored clinically; none of these were recorded among the infant’s complications during the treatment period. Within 3 days of starting propranolol therapy, ascitic production decreased dramatically (Figure 1), continuous octreotide infusion was progressively reduced and discontinued on DOL 61, and, once drainage had remained minimal for a sustained period, propranolol was gradually tapered and discontinued on DOL 68.

Enteral feeding with a formula low in long-chain triglycerides (MCT-based; Basic F, Nutricia, Milano, Italy) was first attempted on DOL 12 but discontinued on DOL 14 during an intercurrent infectious episode; after a further brief attempt on DOL 15–19, feeding was suspended again and definitively restarted on DOL 49, reaching full enteral feeds by DOL 63.

Serial immunoglobulin testing revealed generalized hypogammaglobulinemia consistent with lymphatic protein loss (IgG 2.06–2.64 g/L, IgA < 0.02 g/L, IgM 0.23–0.28 g/L on DOL 4 and 9), for which intravenous immunoglobulin was administered at a dosage of 500 mg/kg on DOL 20, 21, and 40; a persistently low IgA (0.07 g/L on DOL 73) subsequently prompted immunology consultation, which considered this more likely to represent transient hypogammaglobulinemia of infancy than a selective IgA deficiency; concurrent absolute blood lymphocyte counts were low (1.84 × 10^9^/L on DOL 0 and 2.22 × 10^9^/L on DOL 9, both below the age-adjusted reference range), consistent with lymphopenia accompanying the lymphatic protein loss. The infant developed two episodes of catheter-related sepsis: Staphylococcus epidermidis bacteremia on DOL 13, treated with intravenous vancomycin and gentamicin with clinical improvement and a subsequently negative control blood culture; and Staphylococcus capitis bacteremia on DOL 36, treated with intravenous antibiotics, with cerebrospinal fluid analysis negative for meningitis. Propranolol was administered without interruption through both episodes. Inotropic support was never required. The infant was discharged on DOL 93 in stable condition, with ultrasonographically confirmed resolution of ascites maintained through discharge and without further therapy for chylous ascites.

A postnatal comparative genomic hybridization array was repeated and resulted negative. Since the ascites had resolved and no additional clinical concerns were present, further genetic testing was deemed unnecessary; genetics consultation recommended clinical monitoring over time, with repeat genetic evaluation only if the ascites failed to resolve or recurred. Cranial ultrasound, and subsequently brain MRI at DOL 54, identified punctate white matter injury and evidence of prior low-grade germinal matrix/intraventricular hemorrhage; neurodevelopmental examination at DOL 65 was within the normal limits for age. At discharge, a structured post-discharge follow-up program was scheduled, including preterm infant follow-up, pediatric cardiology (echocardiogram and electrocardiogram), audiology, and neurovisual assessment; serial abdominal ultrasound had confirmed no recurrence of ascites through the last examination prior to discharge. A completed CARE checklist is provided as Supplementary Material.

## 3. Review of the Literature

A review of the literature identified one case report and two case series, including a total of nine infants with lymphatic disorders treated with propranolol [10,11,12,13].

Relevant findings are summarized in Table 1. The median (interquartile range) gestational age was 34^+4^ (31^+2^–37^+2^) weeks^+days^. Underlying conditions varied, but all neonates failed to respond to conservative therapy or octreotide and were subsequently treated with propranolol. The duration of treatment ranged from 10 to 76 days with doses between 0.3 and 6 mg/kg/day. All infants achieved complete resolution and were discharged. In two cases, maternal propranolol administration during pregnancy, at dosage of 20–40 mg four times daily, was sufficient, and neonatal therapy was not required.

## 4. Discussion

We reported the successful use of propranolol in a preterm infant with idiopathic congenital chylous ascites that did not improve with standard nutritional management and octreotide.

Chylous ascites results from impaired intra-abdominal lymphatic drainage, leading to the accumulation of lymphatic fluid in the peritoneal cavity. Third-space lymphatic leakage results in the loss of several components within it, such as lymphocytes, albumin, immunoglobulins, coagulation factors, fat-soluble vitamins, enzymes, and electrolytes. These losses cause metabolic and immunological alterations, making prompt and effective management essential [14,15]. Recent evidence further supports the immunological consequences of lymphatic fluid loss, showing altered lymphocyte and immunoglobulin profiles in neonates with chylothorax [16].

Conservative treatment aims to reduce lymph production through fasting and total parenteral nutrition, which bypasses the gastrointestinal tract and minimizes chyle flow. Subsequent cautious refeeding with defatted human milk or formulas low in long-chain triglycerides and enriched with MCT is recommended. Despite these strategies, clinical improvement may be slow and not always achieved [9,15].

Octreotide, a somatostatin analog with a longer half-life than native somatostatin, is often used as second-line therapy. It reduces lymph production by decreasing splanchnic and hepatic blood flow and inhibiting gastrointestinal fat absorption. However, its use may be limited by adverse effects such as hypo- or hyperglycemia, gastrointestinal intolerance, necrotizing enterocolitis, bradycardia, and hypotension [17,18].

Propranolol, a non-selective beta-adrenergic receptor antagonist, has emerged as a promising alternative in refractory cases. Its mechanisms may include splanchnic vasoconstriction, leading to reduced portal venous pressure and lymphatic flow, inhibition of lymphangiogenesis via suppression of vascular endothelial growth factor-A, -C, and -D, and modulation of β_2_-adrenergic receptor pathways that are upregulated in lymphatic malformations. These remain proposed rather than established mechanisms in congenital chylous ascites specifically; propranolol may also exert anti-inflammatory effects through the modulation of macrophage activity and enhance lymphatic vessel contractility, potentially further supporting lymph clearance. Importantly, propranolol has a well-documented safety profile in the treatment of infantile hemangiomas; however, preterm and critically ill neonates with sepsis, prolonged fasting, or parenteral nutrition may carry different risks, particularly hypoglycemia and hemodynamic instability, and safety data specific to this population remain limited [10,11,19,20].

In our patient, propranolol administered orally at a total daily dose of 2 mg/kg was temporally associated with a marked reduction in ascitic drainage within 3 days (Figure 1) and clinical resolution within 37 days. Since propranolol and octreotide were co-administered for about 30 days, and octreotide was discontinued one week before propranolol, the therapeutic effect cannot be attributed surely and exclusively to propranolol. Plausible alternative or contributing explanations include a delayed response to octreotide, which had already been at maximum dose for several days when propranolol was added; the natural fluctuating course of congenital chylous ascites, including spontaneous improvement; and the cumulative effect of ongoing fasting, continued peritoneal drainage, and dietary modification. Nevertheless, given that octreotide alone had not led to improvement over the preceding five days at maximum dose, the temporal association between propranolol initiation and the subsequent decline in drainage suggested a potential beneficial role of this drug.

Among the nine previously reported infants, only two (both reported by Mitchell et al.) had chylous ascites specifically, while the remaining cases involved hydrops fetalis, pleural effusion, or mixed chylothorax/chylous ascites presentations; the two chylous-ascites-specific cases achieved complete resolution within 66 and 114 days of propranolol dosed at 1 mg/kg/day, a course broadly comparable to ours. Despite the variability of the pharmacological dosage used and the different duration of therapy to obtain clinical resolution across the wider group, the existing reports would seem to agree with our observation.

For truly refractory cases not responding to at least 3–4 weeks of conservative and pharmacological therapy, alternative options include dedicated lymphatic imaging and invasive interventions such as thoracic duct ligation, pleurodesis, thoracic duct embolization, or lymphovenous anastomosis, and where a discrete lymphatic malformation is identified, targeted therapies such as sirolimus. It should be noted that propranolol does not resolve chylothorax or chylous ascites in all cases: a case of congenital chylothorax refractory to thoracic duct ligation, octreotide, and propranolol, ultimately requiring chemical pleurodesis, has been reported [2]. Predictors of response to propranolol remain undefined. This report has several limitations. First, conclusions cannot be drawn from a single case. Second, propranolol and octreotide were co-administered, precluding isolation of propranolol’s specific contribution. Third, the diagnosis relied on leukocyte count and mononuclear cell predominance meeting our institutional threshold and the fluid’s macroscopic (“citrine”) appearance; a same-day ascitic-to-serum triglyceride ratio (0.68) did not meet the classical biochemical thresholds for chylous fluid, attributable to the fasting state, and chylomicrons were not assayed, so the diagnosis was clinically consistent but not confirmed on strict biochemical grounds. Fourth, echocardiography excluded a structural cardiac cause and abdominal Doppler incidentally confirmed portal vein patency, but dedicated lymphatic imaging was not performed, so a structural lymphatic anomaly cannot be entirely excluded. Fifth, the underlying etiology remains uncertain. Sixth, the reviewed literature is heterogeneous and subject to potential publication bias, as unsuccessful cases are less likely to be reported. Finally, follow-up duration was limited. Nonetheless, these observations may enrich the limited existing literature and support the rationale for multicenter registries or prospective case series to clarify propranolol’s role in neonatal lymphatic disorders.

## 5. Conclusions

The clinical course of our infant suggested a possible beneficial role in the salvage therapy of idiopathic chylous ascites, even though it was difficult to determine the exact contribution of propranolol versus the combined action of propranolol and octreotide, since both overlapped for one month of treatment. Evidence on the use of propranolol in neonatal lymphatic disorders remains limited, but the existing reports highlighted its potential efficacy. Given the rarity of these conditions, prospective multicenter clinical trials are warranted to establish efficacy, optimal dosing, and treatment duration, mainly considering that no propranolol-related adverse events were observed during treatment in this patient.

## Figures and Tables

**Table 1:** Case report and case series about use of propranolol in neonatal lymphatic flow disorders.

Author (Year)	GA (w/d)	Prenatal Treatment	Diagnosis	Treatment Before Propranolol	Propranolol Dose (mg/kg/day)	Day Start Propranolol	Time to Response (days)	Outcome
Liviskie et al. (2020) [10]	34/4	/	Hydrops fetalis in trisomy 21	TPN, paracentesis	0.3	16	26	Complete resolution
Mitchell et al. (2021) [11]	33/6	/	Idiopathic nonimmune hydrops	TPN, octreotide	1.5 × 4	4	25	Complete resolution
Mitchell et al. (2021) [11]	22/4	/	Chylous ascites and chylothorax post PDA ligation at 29 PMA	TPN, paracentesis, octreotide	1 × 4	52	66	Complete resolution
Mitchell et al. (2021) [11]	23/3	/	Chylous ascites post PDA ligation at 29 PMA	TPN, paracentesis	1 × 4	52	114	Complete resolution
Mitchell et al. (2021) [11]	37/5	/	Postoperative chiloascites at 38 days	TPN, paracentesis	0.75 × 4	39	115	Complete resolution
Handal-Orefice et al. (2023) [13]	37/0	Thoracentesis and maternal medication with propranolol 40 mg × 4 at 19 GA	Idiopathic pleural effusion	/	/	/	/	Complete resolution at birth
Handal-Orefice et al. (2023) [13]	39/3	Thoracentesis and maternal medication with propranolol 40 mg × 4 at 22 GA	Idiopathic nonimmune hydrops	/	/	/	/	Complete resolution at birth
Handal-Orefice et al. (2023) [13]	34/3	Thoracentesis	Idiopathic nonimmune hydrops	Thoracentesis, TPN, MCT formula, octreotide	2	108	137	Complete resolution
Handal-Orefice et al. (2023) [13]	34/0	Thoracentesis	Idiopathic nonimmune hydrops	Thoracentesis, TPN, MCT formula, octreotide	0.3–1	22	57	Complete resolution

MCT—medium chain triglycerides, PDA—patent ductus arteriosus, PMA—postmenstrual age, TPN—total parenteral nutrition.

## Data Availability

The original contributions presented in the study are included in the article/Supplementary Material, further inquiries can be directed to the corresponding authors.

## Supplementary Materials

The following supporting information can be downloaded at: https://www.mdpi.com/article/10.3390/children13091141/s1, CARE Checklist—Propranolol as Salvage Therapy for Refractory Congenital Chylous Ascites in a Preterm Neonate: A Case Report and Review of the Literature.

## Abbreviations

The following abbreviations are used in this manuscript:

TPN	Total parenteral nutrition
MCT	Medium chain triglyceride
DOL	Day of life
CPAP	Continuous positive airway pressure
IgA	Immunoglobulin A
VEGF	Vascular endothelial growth factor
PMA	Postmenstrual age
PDA	Patent ductus arteriosus
