# Supplementary material for: Propranolol as Salvage Therapy for Refractory Congenital Chylous Ascites in a Preterm Neonate: A Case Report and Review of the Literature
Source: Children (Basel). 2026 Aug 26;13(9):1141. doi: 10.3390/children13091141 (PMC13605849; doi:10.3390/children13091141)
Supplement: Supplementary file 1 [file children-13-01141-s001.zip › children-4461201-supplementary.pdf]

CARE Checklist – Propranolol as Salvage Therapy for Refractory Congenital Chylous Ascites in a Preterm Neonate: A Case Report and Review of the Literature

Topic	Item	Checklist item description	Reported in manuscript
Title	1	The diagnosis or intervention of primary focus followed by "case report"	Title page – "Propranolol as Salvage Therapy for Refractory Congenital Chylous Ascites in a Preterm Neonate: A Case Report and Review of the Literature"
Key words	2	2–5 key words identifying diagnoses/interventions, including "case report"	Keywords section: congenital chylous ascites; propranolol; beta-adrenergic blocker; neonate; conservative therapy; case report
Abstract	3a	What is unique about this case and what does it add?	Abstract, Background/Objectives
Abstract	3b	Main symptoms and/or important clinical findings	Abstract, Results
Abstract	3c	Main diagnoses, therapeutic interventions, and outcomes	Abstract, Results
Abstract	3d	Conclusion – main take-away lesson(s)	Abstract, Conclusions
Introduction	4	1–2 paragraphs summarizing why this case is unique, may include references	Introduction
Patient Information	5a	De-identified patient specific information	Case Report
Patient Information	5b	Primary concerns and symptoms of the patient	Case Report
Patient Information	5c	Medical, family, and psychosocial history, including relevant genetic information	Case Report (prenatal history, genetic testing)
Patient Information	5d	Relevant past interventions and their outcomes	Case Report (amnioreduction, fetal paracentesis)
Clinical Findings	6	Significant physical examination (PE) and important clinical findings	Case Report
Timeline	7	Historical and current information organized as a timeline (figure or table)	Case Report narrative; Figure 1 (daily ascitic drainage and concurrent therapies, DOL 0–34)
Diagnostic Assessment	8a	Diagnostic methods (PE, laboratory testing, imaging, other)	Case Report (fluid biochemistry, cytology, ultrasound, echocardiography, genetic testing)
Diagnostic Assessment	8b	Diagnostic challenges (e.g., access to testing, financial, cultural)	Discussion, Limitations paragraph
Diagnostic Assessment	8c	Diagnostic reasoning, including other diagnoses considered	Case Report; Discussion (differential considerations, genetic testing to exclude syndromic causes)
Diagnostic Assessment	8d	Prognostic characteristics, where applicable	Discussion
Therapeutic Intervention	9a	Types of therapeutic intervention (pharmacologic, surgical, preventive, self-care)	Case Report (TPN, octreotide, oral propranolol, IVIG, albumin, antibiotics)
Therapeutic Intervention	9b	Administration of therapeutic intervention (dosage, strength, duration)	Case Report
Therapeutic Intervention	9c	Changes in therapeutic intervention, with rationale	Case Report (octreotide dose titration; propranolol initiation, tapering, discontinuation)
Follow-up and Outcomes	10a	Clinician- and patient-assessed outcomes, where appropriate	Case Report; Discussion

Follow-up and Outcomes	10b	Important follow-up test results (positive or negative)	Case Report (serial abdominal ultrasound, neuroimaging, immunoglobulin panel)
Follow-up and Outcomes	10c	Intervention adherence and tolerability (how was this assessed?)	Case Report (clinical and vital sign monitoring for propranolol-related adverse effects)
Follow-up and Outcomes	10d	Adverse and unanticipated events	Case Report (sepsis episodes); Discussion (absence of propranolol-related adverse effects)
Discussion	11a	Strengths and limitations of the approach to this case	Discussion, Limitations paragraph
Discussion	11b	Discussion of the relevant medical literature	Review of the Literature; Discussion
Discussion	11c	The rationale for conclusions	Discussion
Discussion	11d	Primary take-away lessons (without references)	Conclusion
Patient Perspective	12	The patient/family should share their perspective on the treatment(s) received, where appropriate	Not applicable – neonatal patient; parental perspective not formally collected
Informed Consent	13	Was informed consent obtained? (Provide if requested)	Written informed consent obtained from the patient's parents – see Informed Consent Statement
